# A 3-min weighted vests re-warmups induce sprint performance enhancements at the start of the second half of a soccer match-play

**DOI:** 10.3389/fphys.2023.1173991

**Published:** 2023-07-06

**Authors:** Mohamed Amine Ltifi, Olfa Turki, Ghazi Racil, Alin Larion, Mohamed Souhaiel Chelly, Helmi Ben Saad, Riadh Khalifa, Karim Chamari, Johnny Padulo

**Affiliations:** ^1^ Higher Institute of Sport and Physical Education of Ksar-Said, University of La Manouba, Tunis, Tunisia; ^2^ Research Laboratory (LR23JS01) “Sport Performance, Health & Society”, Higher Institute of Sport and Physical Education of Ksar Said, University of Manouba, Tunis, Tunisia; ^3^ Faculty of Physical Education and Sport, Ovidius University of Constanta, Constanta, Romania; ^4^ Faculty of Medicine of Sousse, Hospital Farhat HACHED of Sousse, Research Laboratory Heart Failure, University of Sousse, Sousse, Tunisia; ^5^ Aspetar, Orthopaedic and Sports Medicine Hospital, FIFA Medical Centre of Excellence, Doha, Qatar; ^6^ Department of Biomedical Sciences for Health, Università Degli Studi di Milano, Milan, Italy

**Keywords:** post-activity performance enhancements, rating of perceived exertion, sprint performance, team sport, match play

## Abstract

**Purpose:** This study aimed to investigate the effect of re-warm-up (RWUP) activities consisting of three sets of 15-m sprints with weighted vests on 20-m sprint performance after the break.

**Methods:** Using a randomized, and counterbalanced cross-over design, twenty U15 elite soccer players performed four RWUP trials which consisted of 15-min of passive rest (control: CONT), 3 × 15-m sprint (RW0%), sprinting with a vest-loaded at 5% of body-mass (BM) (RW5%), sprinting with a 10% BM vest (RW10%). The 3-min RWUP protocols started 10-min after the commencement of the 15-min between-halves break and concluded 2-min before its end. During each testing session, the participant’s RPE scores, and 20-m sprint performance were registered before the first half, and at the end of the break of the simulated match-play.

**Results:** Significant time effects [F (1.72) = 54.37, d = 1.88, *p* < 0.001; F (1.72) = 9.07, d = 0.77, *p* = 0.003], and condition effects [F (3.72) = 11.81, d = 1.53, *p* < 0.001; F (3.72) = 7.36, d = 1.21, *p* = 0.003] were observed for RPE scores and 20-m sprint performance, respectively. Significant condition-by-time interactions were found for RPE scores (*p* < 0.003, d = 0.54), and 20-m sprint performance (*p* < 0.002, d = 0.70). Contrast analysis showed significantly higher RPE scores (*p* < 0.001, d = 1.55), and improved 20-m sprint performance (*p* = 0.0004, d = 1.19) in the RW10% condition compared to all other conditions.

**Conclusion:** Sprinting for 3-min with a 10% body-mass vest resulted in the highest RPE scores and the most substantial enhancements in 20-m sprint performance. Young elite soccer players should incorporate 10% body-mass weighted vests in their re-warm-ups to boost post-break sprint performance.

## 1 Introduction

In football (soccer), the players warm-up before matches, but usually not during the break separating the two-halves (González-Devesa et al., 2021). The traditional 15 min of passive break in-between halves, reduces the efficacy of the pre-competition warm-up (Russell et al., 2015b). During the initial stages of the second half of team-sports competition, a decline in athletic physical performance (Russell et al., 2015b; Hammami et al., 2018; Silva et al., 2018; González-Devesa et al., 2021), and a potential increase in the player’s risk of injury (Rahnama et al., 2002) have been reported. During a match, the amount of sprinting is associated with the match outcome (Chmura et al., 2018). Mohr et al. (2004) also showed that players performed a lower amount of high intensity running during the first 15 min of the second half compared to the first half’ same period of time. Thus, it is of utmost importance to develop short active re-warm-up (RWUP) strategies that are in accordance with the increasingly high demands of the sporting activity (González-Devesa et al., 2021; Manuel Clemente et al., 2021)^.^

Concerning the 15 min passive rest taken at half time, different RWUP strategies including moderate-intensity run, whole-body vibration, and lower-body resistance exercise have proved their superiority in preventing the decrements in sprint ability (Mohr et al., 2004; Lovell et al., 2013; Zois et al., 2013). Several RWUP reports supported the use of different durations of low, moderate and high-intensity RWUP activities during the half-time break (Hammami et al., 2018; Silva et al., 2018; González-Devesa et al., 2021). However, and to date the implementation of active RWUPs in actual competition settings remains conflictual (only 58% of coaches use active re-warm-up) due to the limitation of time and the ecological aspects of the protocols (Towlson et al., 2013).

Previous studies have shown beneficial changes in performance after both 5 min (Lovell et al., 2007; Lovell et al., 2013) and 7 min (Mohr et al., 2004; Yanaoka et al., 2018a) RWUP protocols, when compared to a passive break. Nevertheless, this duration might not be applicable during the competition as the coaches need a substantial amount of time to speak to their players in addition to the time needed by the players to recover from the first half efforts (Russell et al., 2015b). In this prospective, only 1 min duration, high-intensity cycling (Yanaoka et al., 2020a), and running (Yanaoka et al., 2020b) at 90% of the maximal oxygen uptake showed improved cycling intermittent-sprint protocol in 12 active males (Yanaoka et al., 2020a), and improved Loughborough Intermittent Shuttle Test performance, core temperature, muscle activation, and heart rate in 12 male amateur team sports players (Yanaoka et al., 2020b). Some reviews proposed a theoretical model of RWUP and emphasized that, in actual competition matches; the players typically have no more than about 3 min available for an active RWUP (Towlson et al., 2013; Russell et al., 2015b). Recent reports have indicated that a 3-min cycling based RWUP was as effective as a 7-min RWUP for improving intermittent sprint performance (Yanaoka et al., 2018a). Similar duration of RWUP protocols that consisted of 3 min cycling at low [30% of maximal oxygen uptake (VO_2max_)] (Yanaoka et al., 2018b; Yanaoka et al., 2020a), moderate (60% of VO_2max_) (Yanaoka et al., 2018b), or high (90% of VO_2max_) (Yanaoka et al., 2020a) intensities have been recently investigated. Nonetheless, on the basis that it mimics the activity pattern and the load executed in team sports, these studies used intermittent cycling exercise as a first half, which is far from simulating the actual football game demands (Yanaoka et al., 2018a; Yanaoka et al., 2018b; Yanaoka et al., 2020b)^.^

High-intensity, short-duration soccer-specific conditioning activities (such as sprints and jumps) may act as an ergogenic aid by acutely potentiating subsequent 20-m sprint performance in soccer players (Brink et al., 2022). Postactivation potentiation (PAP) is the commonly acknowledged phenomenon by which muscular performance is acutely and temporarily enhanced because of muscular contractile history (Hodgson et al., 2005). After a conditioning contraction, the time course of post-activation potentiation (PAP) closely corresponds to the time course of MRLC phosphorylation (Blazevich and Babault, 2019). Therefore, PAP exhibits a short-lived duration (seconds to 5 min) (Blazevich and Babault, 2019). Besides, Recent studies have shown that enhancement in voluntary force production may be partly due to changes in muscle temperature, muscle/cellular water content, and muscle activation through a phenomenon named post-activation performance enhancement (PAPE) (Cuenca-Fernández et al., 2017; Blazevich and Babault, 2019). PAPE demonstrates a delayed onset, taking several minutes to manifest (6–10 min) and exhibiting a prolonged duration of over 15 min (Blazevich and Babault, 2019).

The use of resisted short and high-intensity RWUP activities may be more advantageous than previously investigated with unloaded short and high-intensity (Yanaoka et al., 2018b; Yanaoka et al., 2020a) RWUPs in elite football players. A study conducted by Zois et al. (2013) demonstrated the superiority of a 5 maximum repetitions (RM) leg-press RWUP strength exercise at enhancing subsequent sprint performance. However, one should consider the lack of practicality (*e.g.*, lack of equipment and facilities) that could prevent from implementing this RWUP strategy during matches (Zois et al., 2013). Thus, a time efficient RWUP with ecological equipment setting is strongly needed.

For soccer players and coaches, the ability to enhance soccer-related skills after the re-warm-up phase, without the need for specialized equipment or facilities, would provide significant advantages. A frequently employed conditioning activity among athletes involves performing the sport-specific skill during the warm-up while wearing weighted vests (Maloney et al., 2014; Turki et al., 2020). The aim of these strategies is to induce acute enhancements in neuromuscular function and promote increased strength and power production during subsequent exercises after the warm-up (Zois et al., 2011). Past reports (Maloney et al., 2014) showed enhancements in change of direction speed (up to the 6th minute post warm-up) after using 5% and 10% of body mass (BM) weighted vests in professional badminton players. However, implementing a dynamic warm-up with a 5% loading did not yield significant benefits in terms of increasing lower extremity power output among high school football players (Reiman et al., 2010). Another study reported better total and peak repeated change of direction times up to the 8th minute following 5%, 10%, and 15% weighted vests (worn during the final 3 exercises of the specific warm-up) preconditioning activities in 19 young male soccer players, when compared with unloaded condition (Turki et al., 2020).

To date, and to the better of the authors’ knowledge, no consensus has been reached on an optimal loaded RWUP strategy or protocol to be applied during the break in soccer matches. In this prospective, the use of very short and weighted vests RWUP strategy during the break could be advantageous for elite players to acutely optimize soccer related performances compared with active unloaded RWUP activities and therefore, deserves to be explored. Thus, the aim of this study was to investigate the effect of re-warm-up (RWUP) activities consisting of three sets of 15-m sprints with vests weighted at 5% of body mass (RW5%), and 10% of body mass (RW10%) on 20-m sprint performance, and ratings of perceived exertion (RPE) at the end of the break of a simulated match in young elite male soccer players. “It was hypothesized that: *H*_*1*_: RW10% would lead to greater changes in the RPE scores and 20-m sprint performance when compared with RW0%, RW5%, and control (CONT); *H*_*2*_: RW10% would yield greater improvements in the RPE scores, and 20-m sprint performance compared to RW0% and RW5%; and *H*_*3*_: RW10% would yield greater improvements in the RPE scores and 20-m sprint performance compared to CONT.”

## 2 Material and methods

### 2.1 Participants

Twenty top-level under U15 male soccer players (Age: 14.55 ± 0.51 years; body height 1.73 ± 0.06 cm; body mass 61.20 ± 7.57 kg) from the same club (National league-1st club) voluntarily participated in this study. All participants had a background of at least 8 years of systematic soccer training involving ∼5 training sessions per week, with a soccer match on the weekend throughout the ∼10 months soccer seasons. Prior to joining this club, all of these players underwent extensive training at a specialized professional academy, which encompassed various aspects such as technical skills, tactical understanding, mental preparation, and physical conditioning. The primary focus of this academy was to identify and develop talent in young players for the club.

None of the players reported having had any substantial musculoskeletal disorder during the 3 months prior to the study. Verbal and written informed consent from legal representatives and verbal assent from children were obtained after an explanation of the experimental protocol and its potential benefits and harms. The study protocol was approved by the local Institutional Review Committee of the Faculty of Medicine of Sousse, Tunisia (CEFMS 124/2022).

### 2.2 Experimental design

A randomized and counterbalanced, repeated-measures design was used to determine whether weighted vests RWUP is more advantageous for young soccer players to use during the football match half-time break. Vest weighted RWUP were compared with active unloaded or passive rest RWUPs to investigate if it would induce subsequent 20-m sprint post-activity performance enhancements (PAPE) during the early phase of the second half of a soccer match play. Participants were invited on six different occasions to undertake two familiarization and four testing sessions.

The first orientation session was devoted to collecting anthropometric data from participants (*i.e.*, age, body mass (BM), stature, and percentage of body fat), and to familiarize them with the RPE measurements. During the second familiarization session, each participant was familiarized with the RWUP procedures, and the 20-m sprint test to minimize any learning effect even though the players were used to routine sprint testing.

**FIGURE 1:**
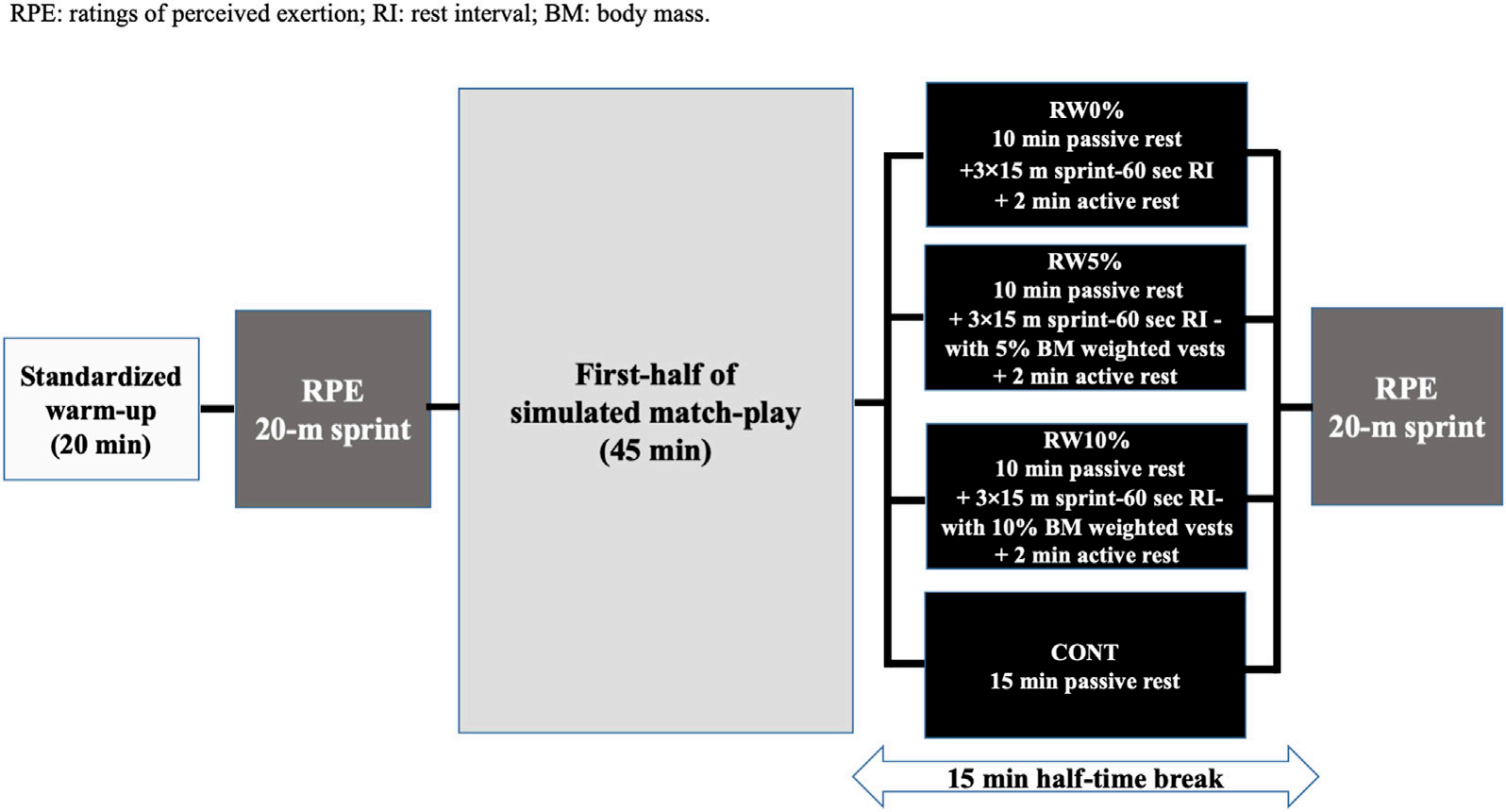
Schematic representation of the experimental design.

Each testing session began with the same 20 min standardized soccer warm-up followed by baseline assessments of the participant’s RPE and 20-m sprint performance. Further methodological details are provided below. The participants were then required to play 45 min of a simulated match (first half) before undertaking in a counterbalanced and randomized order and in different days, four experimental conditions involving four different combinations: *(i)* 10 min seated rest +3 × 15-m sprint (with 60 s recovery in-between) (RW0%); *(ii)* the same but sprinting with a weighted vest loaded at 5% BM (RW5%); *(iii)* the same but sprinting with a weighted vest loaded at 10% BM (RW10%), and *(iv)* 15 min passive rest control condition (CONT) as shown in Figure 1.

The RW0%, RW5%, RW10% featured an active component, which was initiated at the 10th minute mark of the break and consisted of three minutes of either loaded or unloaded 3 × 15-m sprints. The 5%, and 10% of the participant’s body mass loads were chosen based on Turki et al. (2020) findings. The authors demonstrated that a dynamic weighted vests (5%, 10%, and 15% BM) warm-up increases RCOD performance up to the 8th minute post-warm-up intervention in young soccer players. Following the completion of the unloaded or loaded conditioning sprints, players had a two-minute period of active rest, during which they walked to the location for the RPE assessment before attaining the location of the 20-m sprint test assessment. Therefore, a period of 4-5 min separated the end of the 3 × 15-m loaded or unloaded conditioning sprints and the completion of the 20-m sprint test. During the four experimental sessions, the subject’s ratings of perceived exertion scores (RPE), and the 20-m sprint performance were registered at two different time points: *(i)* after the standardized warm-up; and *(ii)* at the end of the half-time break (Figure 1). The first half of the simulated matches (45 min of play) were held in the presence of a referee and a linesman.

The RW0%, RW5%, RW10% re-warm-up interventions attempted to reunite all the conditions to fulfill to the athlete’s needs during the 15 min break: (i) the first 10 min of passive rest were designed to enable the players to relax mentally from the cognitive and physical demands of the first half of match-play, receive eventual medical treatment, rehydrate, and receive tactical instruction and coach feedback; (ii) the following 3 min of high intensity activities (3 × 15-m sprint) were designed to induce PAPE in subsequent 20-m sprint performance at the end of the 15 min half-time break. This typical and novel structure was chosen based on previous recommendations (Russell et al., 2015a) who proposed a theoretical model of RWUP and emphasized that, in actual matches, the players typically have about 3 min available for an active RWUP.

The 20-m sprint test was performed in an outdoor soccer pitch (4G artificial turf field). The 20-m distance was chosen because it represents the mean sprint distance in field-based team sports assessment (Sim et al., 2009), and because this distance was part of the participants’ regular fitness testing battery. Sprint ability in football is commonly evaluated through the 20-m sprint test (Franco-Márquez et al., 2015; Nikolaidis et al., 2016), which requires participants to cover this distance as quickly as possible. Although other distances, such as the 40-m sprint test (Le Gall et al., 2002), have been utilized to assess sprint abilities, it has been suggested that distances longer than 20-m are not as specific to the sport (Barnes et al., 2014).

Each player performed three maximal 20-m sprints intersected by a ∼3 min recovery period during which the participants walked back to the starting line and waited for the next trial. The best of the three trials was retained for analysis. The participants began each trial in their own time, from a standing start 0.5 m behind the first timing gate. The participants were asked to continue sprinting at maximum effort until the finishing timing gate. Participants were strongly motivated and encouraged to give their utmost effort and strive for maximum performance in each trial of the 20-m sprint test. Time was recorded using photocell gates (Brower Timing Systems, Salt Lake City, UT; accuracy of 0.01 s) placed 0.4 m above the ground.

Our study design, which involved actual matches, is a novel approach that offers a higher level of realism compared to previously published RWUP study protocols. Therefore, it was not feasible to implement equal training loads for all athletes. Playing a match under real conditions serves as a limit for the effort load that will be demanded of the players. The offensive and defensive actions performed during actual game situations, which include active rest periods between actions, cannot be predetermined. Throughout the four testing sessions, the participants consistently played in the same position during the first half of the matches. There were no substitutions made during any of the four sessions.

During the four testing sessions, wind speed was measured in meters per second in either a positive (tailwind) or negative (headwind) direction. The RPE (Borg scale) was used with a scale of 0–10 as previously reported (Foster et al., 2001)^.^ The RPE scores were verbally provided by each player and recorded on a spreadsheet.

This study design was primarily focused on enhancing ecological validity, which involves prioritizing the development of realistic and effective strategies for athletes and coaches to actually apply during matches, thereby increasing the applicability of RWUPs on the pitch. To enhance the practical relevance of this study, it was crucial to ensure that its design apply similar durations and sequences (first half and break) as those used in real competition. The total timing allocated to the RPE and sprint measurements (data collection) was about 10- to 11 min. In Mohr et al. (2004) study, the players performed a sprint test immediately after the first half. Nevertheless, our strategic decision was to align as closely as possible with the real-world football conditions, which involved adhering to the actual match timings allocated to players during the competition. Moreover, collecting data at the end of the first half would likely have imposed an additional strain on the players, exceeding the physical demands typically associated with a 45 min match, potentially modifying the effects of the RWUP on 20-m sprint performance and RPE scores collected at the end of the break.

The experiments took place in Bardo, Tunisia, between August and September 2022. Data collection occurred at approximately the same time of day, specifically between 9:00 p.m. and 11:00 p.m., which coincided with the regular training time for that period. The data collection venue was a soccer stadium. (Temperature: 33.75°C ± 1.26°C, humidity 43.75% ± 6.18% and wind 18 ± 11.46 km h^−1^). A schematic representation of the experimental design is shown in Figure 1.

### 2.3 Statistical analyses

Data are presented as means and standard deviations (SD) and normality was assessed and confirmed using the Shapiro-Wilk test. The data were then analyzed using a 4 condition: (*i.e.*,; RW0%, RW5%, RW10%, and CONT) by 2 time: (*i.e.*,; pre-, post rewarm-up interventions) analysis of variance (ANOVA) for repeated measures. Where the assumption of sphericity was violated, Greenhouse-Geisser correction was used to interpret the results. Where any significant differences were found, *post hoc* pairwise comparisons (Tukey) were used. Additionally, effect sizes (ES) were determined from ANOVA by converting partial eta-squared to Cohen’s d, in accord with Cohen (1988). Moreover, within-group ES were computed using the following equation: ES = (mean post–mean pre)/SD. ES were considered to be either “trivial” (<0.2) “small” (>0.2–0.6), “moderate” (>0.6–1.2), “large” (>1.2–2), or “very large” (>2) (Hopkins et al., 2009).

For the varying conditions, i.e., RW0%, RW5%, RW10% and CONT, contrast analysis (Haans, 2008; Hervé and Williams, 2010) were carried out to specifically test the following hypotheses; H1) RW10% would lead to greater changes in the RPE scores and 20-m sprint performance compared with all other conditions (RW0%, RW5%, CONT), H2). RW10% would yield greater improvements in the RPE scores and 20-m sprint performance compared to RW0% and RW5%, H3) RW10% would yield greater improvements in the RPE scores and 20-m sprint performance when compared with CONT. Accordingly, three contrasts were computed. First, we compared the RW10% condition vs. RW0%, RW5% and CON conditions. Second, we compared the RW10% condition vs. RW0% and RW5%. Third, we compared the RW10% condition vs. the CONT condition. This approach yielded a comparison of one (or more) condition (s) vs. the grand mean of the specified contrasts. Indeed, *post hoc* analyses, while useful, do not yield sufficient insight into multiple levels or detailing patterns in response; whereas contrast analysis allows researchers to test theory-driven expectations directly against empirically derived group or/condition (Rosnow and Rosenthal, 1995; Rosnow and Rosenthal, 1996). The level of significance was set at *p* < 0.05. The statistical analysis was carried out using IBM SPSS (version 25).

## 3 Results

All participants completed the study according to the study design and methodology. Participants attended all testing sessions and none reported any exercise- or test-related injury. The reliability of the measurements showed an ICC of 0.609 (Good) for 20-m sprint performance.

### 3.1 ANOVA and *post hoc* results

There was a statistically significant main time effect for RPE and 20-m sprint performance [F (1.72) = 54.37, d = 1.88, *p* < 0.001; F (1.72) = 9.07, d = 0.77, *p* = 0.003 respectively]. In addition, there was a statistically significant main condition effect for RPE scores and 20-m sprint performance [F (3.72) = 11.81, d = 1.53, *p* < 0.001; F (3.72) = 7.36, d = 1.21, *p* = 0.003, respectively]. Significant condition-by-time interactions were found for RPE scores (*p* < 0.003, d = 0.54) and 20-m sprint performance (*p* < 0.002, d = 0.70; Table 1). The between-condition *post hoc* testing highlighted statistically significant differences between RW10% *versus* RW5% and RW0% [*p* < 0.0001 (d = 1.01); *p* < 0.001 (d = 0.87), respectively] for 20-m sprint performance. The within-group *post hoc* tests showed higher RPE scores and better 20-m sprint performance in the RW10% condition, compared to RW5%, and RW0%, respectively.

**TABLE 1:** Results of the two-way analysis of variance (ANOVA) for repeated measures.

Variables	Conditions	Pre-intervention	Post-intervention	ANOVA *p*-value (Cohen’s d)		
				Time	Condition	Condition x time
RPE	RW10%	1.55 ± 0.48	5.10 ± 1.10	0.001 (1.88)	0.001 (1.53)	0.003 (0.54)
	RW5%	1.35 ± 0.43	3.95 ± 0.72			
	RW0%	1.23 ± 0.30	2.68 ± 0.81			
	CONT	1.51 ± 0.47	1.13 ± 0.27			
20-m sprint time (s)	RW10%	3.17 ± 0.12	2.97 ± 0.13	0.003 (0.77)	0.003 (1.21)	0.002 (0.70)
	RW5%	3.12 ± 0.17	2.97 ± 0.11			
	RW0%	3.16 ± 0.14	3.02 ± 0.13			
	CONT	3.13 ± 0.15	3.16 ± 0.11			

Notes: Values are expressed as means and standard deviations (SD), RW10% = 3 × 15-m sprint with vests weighted at 10% of body mass (BM), RW5% = 3 × 15-m sprint with vests weighted at 5% BM; RW0% = 3 × 15-m sprint, CONT = 15-min seated rest.

### 3.2 Contrast analyses

Specific to contrast analyses, all specified hypotheses were tested and are detailed in Table 2. The contrast 1 (RW10% vs. All), indicated that RW10% yielded statistically significant higher RPE scores (*p* < 0.0001, d = 1.55), and better 20-m sprint performance (*p* = 0.0004, d = 1.19). The contrast 2 (RW10% vs. RW5% + RW0%), showed that RW10% yielded statistically significant higher RPE scores and better 20-m sprint performance (both, *p* < 0.0001, d = 2.03 and 1.45 respectively). Finally, the contrast 3 (RW10% vs. CON), showed that RW10% yielded statistically significant higher RPE scores (*p* = 0.04, d = 0.65), and better 20-sprint performance (*p* = 0.014, d = 0.47).

**TABLE 2:** Contrast analysis of the re-warm-up conditions (RW10%, RW5%, RW0%, and CONT) for the RPE scores and the 20-m sprint performance.

		RPE				20-m sprint time			
		*p*	Mean diff	T	SE	*p*	Mean diff	T	SE
RW10%	ALL	<0.0001	0.49	4.92	99.78	0.0004	1.3	3.76	3.63
RW10%	RW5% + RW0%	<0.0001	0.66	6.43	94.68	<0.0001	1.7	4.61	3.73
RW10%	CON	0.04	0.25	2.08	122.2	0.014	4.63	1.49	4.44

Notes: All differences are manifest from the grand means of the specified contrast(s), RW10% = 3 × 15-m sprint with vests weighted at 10% of body mass (BM), RW5% = 3 × 15-m sprint with vests weighted at 5% BM, RW0% = 3 × 15-m sprint, CONT , 15-min seated rest, p: *p*-value, Mean Diff: mean difference, T: t-value, SE: standard error.

## 4 Discussion

We investigated the effect of 3 min weighted vests re-warm-ups (RWUP) on 20-m sprint performance, and ratings of perceived exertion (RPE) at the end of the half-time break of a simulated match play in young elite male footballers. Collectively the main findings indicated significant time and condition effects for RPE scores and 20-m sprint performance, respectively. Contrast analysis showed that all the hypotheses were supported by the results obtained. Considering that the differences in RPE values were not very pronounced between both loaded conditions, RW10% proved to be the most effective RWUP strategy to apply to enhance 20-m sprint performance at the end of the break.

Straight sprinting is the most frequent action during goal situations in professional football matches (Faude et al., 2012). Therefore, improving sprint performance at the start of a second half could have potential benefits for the entire match outcome. The present study may provide new insight regarding the effects of loaded RWUPs in young elite players, which may have beneficial applications for coaches and strength and conditioning (S&C) experts. Past reports explored the effect of dynamic pre-competition warm-up with vests loaded from 2% to 15% BM on athletic performance in 18 high school female athletes (Faigenbaum et al., 2006), 16 athletics’ women (Thompsen et al., 2007), in 8 elite badminton players (Maloney et al., 2014), and in 19 male football players (Turki et al., 2020). However, and to the best of the authors’ knowledge, this study is the first to use weighted vests as a RWUP activity at half-time in football. Zois et al. (2013) reported an improved repeated-sprint ability during the second period of the match following a 5 RM leg press RWUP intervention. However, the lack of practicality of this strategy, due to the lack of gym equipment next to all the football pitches, could prevent for implementing it in real-game settings. The RW10% protocols had a novel approach, and clear advantages in terms of applicability, compared with Zois et al.’s protocol (2013). Wearing loaded vests is clearly easy and quick in terms of practicality and implementation during the half-time break. Consequently, we considered it of upmost importance to propose realistic loaded RWUP activities to enable athletes to initiate a second half with an optimal level of their physical capacities.

The observed enhancement in sprint performance at the end of the break is likely associated with various physiological factors, including elevated heart rate, core and muscle temperature, improved muscle oxygenation, altered blood metabolite response, and increased neuromuscular activity (González-Devesa et al., 2021). In this regard, changes in muscle temperature, muscle/cellular water content, and muscle activation have been pointed to (at least partly) underpin voluntary force enhancement through post-activation performance enhancement (PAPE) (Blazevich and Babault, 2019)^.^ PAPE typically exhibits a delayed onset, with effects taking several minutes (6–10 min) to become apparent. Moreover, PAPE is known to have a prolonged duration lasting over 15 min (Blazevich and Babault, 2019). Previous reports have also indicated that high intensity voluntary conditioning contraction enhances muscle contractile response through a phenomenon called post-activation potentiation (PAP) (Tillin and Bishop, 2009; Blazevich and Babault, 2019). PAP is a well-described phenomenon that enhances muscle force production mainly through an increased myosin light chain phosphorylation (Blazevich and Babault, 2019). After a conditioning contraction, the phosphorylation process occurs rapidly so performance enhancements should be nearly immediate (Tillin and Bishop, 2009; Blazevich and Babault, 2019). However, the process is also reversed rapidly by removal of the phosphate group from the MRLC by myosin light chain phosphatase (Russell et al., 2015a; Russell et al., 2018). This results in a quasi-exponential rate of decline in PAP with a rapid decline in the first ∼28 s and only a small effect present by ∼5 min (Vandervoort et al., 1983; Hamada et al., 2000)^.^ The protocol of this study did not include any additional measurements likely to invalidate or formally affirm the contribution of PAP and/or PAPE. However, it is important to specify that after completing either the unloaded or loaded conditioning sprints, the players had a two-minute period of active rest, during which they walked to the location for the ratings of perceived exertion (RPE) assessment. Subsequently, they proceeded to the location for the 20-m sprint performance test. Therefore, there was a time gap of approximately 4-5 min between the completion of the sprint-based conditioning RWUP activity and the actual 20-m sprint performance assessment. Several variables, such as volume, intensity, training status, and rest period length, influence the magnitude of post-activation potentiation (PAP) and its relationship with fatigue (Tillin and Bishop, 2009; Wilson et al., 2013). Meta-analysis by Wilson et al. (2013) suggests that moderate rest periods (7–10 min) may produce optimal power output after a conditioning activity, while more trained individuals might benefit from shorter recovery times (3–7 min) for greater PAP effects. Consequently, we speculate that the above-mentioned mechanisms underpinning both PAP and PAPE have contributed to this study’s results. It is worth noting that residual post-activation potentiation (PAP) effects might have influenced the early stages of postactivation performance enhancement (PAPE) in the highly trained participants of this study.

Our findings suggest that the potentiating intensity of the re-warm-up stimulus generated by the unloaded and 5% of body mass conditions may not have been sufficient to achieve the same level of potentiation observed in the RW10% condition among the highly-trained young participants of this study. Besides, as greater conditioning contraction volumes are expected to induce greater levels of both PAP and fatigue (Tillin and Bishop, 2009), it is likely that an enhanced 20-m sprint performance could be attained by including more repetitions of 15-m sprints during the re-warm-up phase. It is important to note that in a soccer competitive setting, the time constraints during the between-half break may make it challenging to incorporate a larger number of repetitions of RWUP activities. As an illustration, performing 6 sets of 15-m sprints as a RWUP activity would take a total of 6 min, which may not be feasible during a break in a competition. As shown by the results of this study, it may be more appropriate to manipulate the intensity of the RWUP conditioning activity by using short loaded activities.

The interest of our study design may mainly lie in its ecological validity. To make RWUPs more applicable on the pitch, it has been prioritized to provide athletes and coaches with realistic and valuable strategies for use on match day. Similar to this study protocol, a very limited number of past RWUP published protocols (Mohr et al., 2004) involved football play (Friendly match) as a first half. Of note, the majority of RWUP studies opted for a laboratory tests (i.e., intermittent cycling exercise) as a simulation of a first half on the basis that it simulates the activity pattern and the workload imposed by intermittent team sports (González-Devesa et al., 2021). However, intermittent team sports such as football comprise running, sprinting, changes of directions, physical contact, and other types of exercise. The study protocols using only one type of exercise (cycling) lack similarity to the real football match environment. Thus, it is unknown whether these RWUP protocols will have the same effect on performance during actual matches.

We analyzed the relationship between maturity, peak height velocity (PHV), and 20-m sprint performance in the RW5% and RW10% conditions. Maturity offset varied significantly (−0.175–1.618 years) without a clear correlation to sprint performance. Similarly, sprint times showed considerable variation (2.85–3.24 s) without a consistent trend based on maturity offset. These findings indicate that sprint performance is not solely determined by maturity, as participants with different maturity offsets achieved diverse sprint times.

The results indicated that the most significant increases in RPE scores were observed following the loaded RWUP conditions compared to baseline measures. The greatest enhancements in RPE scores were observed in the RW10% condition. These findings are consistent with previous reports on RWUP, which assessed RPE scores immediately after the break and found that active interventions resulted in higher perceived exertion compared to passive rest (González-Devesa et al., 2021). Gonzalez Deveza et al. (2021) advocated the use of sport-specific activities during the RWUP intervention to facilitate the transfer to the second half of play. The authors also noted that most published RWUP protocols lack ecological validity. As a result, our findings may have significant practical application. Even though weighted vests RWUP activities may generate a higher RPE than passive rest and active unloaded RWUP strategies, it is recommended that highly-trained players incorporate them to enhance their sprint abilities immediately after the break.

Some methodological limitations to our study should be acknowledged. Firstly, this study protocol didn’t involve important physiological measurements such as heart rate, core or muscle temperature, and electromyography to better describe the effect of the RWUP protocols. Secondly, our study protocol has not included a second-half play. Thus, it remains unclear whether the positive effects of the loaded RWUP experimental conditions would have continued throughout the 45 min of the second half. This should be the concern of future studies. Thirdly, the effect of performing the RWUP in different environments is not clear. For example, in cold environments, core and muscle temperatures may decrease to a greater extent than in thermoneutral or hot environments. Finally, as in international matches, the players must return to the changing room after the pre-competition warm-up and passively rest for a period that can reach 20–25 min before the start of the match, a promising research topic may be linked with the implementation of short and high intensity RWUP activities preceding the start of the first half of a match.

Moreover, a recent review (González-Devesa et al., 2021) have advocated the use of combined active and passive heat RWUP strategies. A Passive heating method consisting of immersing to the gluteal fold in a hot bath did not reduce the decrement in performance in seven professional players (Lovell et al., 2007). In addition, both active RWUP and passive heat maintenance methods are effective (Russell et al., 2018). However, the combined method (i.e., 8 min of wearing a survival jacket followed by 7 min of RWUP activities) of this study elicited additive beneficial performance effects in comparison to both alone (Russell et al., 2018). In this regard, combining heated garments (*i.e.*, heated clothing and heating pads) to maintain elevated post-first half muscle temperature and resisted activities could also be the focus of further research.

Finally, as the sustained effect of RWUP on exercise performance is not clear, future research should explore the best combination of volume, intensity, and rest period of very short (1–3 min) loaded RWUP interventions while including physical performance measurements during the course and till the end of a second half of match play.

## 5 Conclusion

Our findings indicate that engaging in RWUP activities, which involved three sets of 15-m sprints with weighted vests loaded at 10% of body mass, resulted in the most significant improvement in 20-m sprint performance at the end of the half-time break. As minimal differences in RPE scores were found between the two loaded RWUP conditions, the RW10% condition emerged as the most effective re-warm-up strategy for enhancing 20-m sprint performance after the break. Given time limitations, it is advisable for sport scientists and coaches to incorporate short bouts of high-intensity running with weighted vests within 10 min of the commencement of a 15-min break.

## Data Availability

The raw data supporting the conclusion of this article will be made available by the authors, without undue reservation.
